# Bis(*N*-isopropyl-*N*-methyl­dithio­carbamato-κ^2^ *S*,*S*′)diphenyl­tin(IV)

**DOI:** 10.1107/S1600536810007415

**Published:** 2010-03-03

**Authors:** Amirah Faizah Muthalib, Ibrahim Baba, Yang Farina, Seik Weng Ng

**Affiliations:** a School of Chemical Sciences, Universiti Kebangbaan Malaysia, 43600 Bangi, Malaysia; b Department of Chemistry, University of Malaya, 50603 Kuala Lumpur, Malaysia

## Abstract

The dithio­carbamate anions in the title compound, [Sn(C_6_H_5_)_2_(C_5_H_10_NS_2_)_2_], chelate to the Sn^IV^ atom, which is six-coordinated in a skew-trapezoidal-bipyramidal geometry. The mol­ecule lies across a twofold rotation axis.

## Related literature

For other diphenyl­tin bis­(dithio­carbamate) compounds, see: Alcock *et al.* (1992 ▶); Farina *et al.* (2001*a* ▶,*b* ▶); Hook *et al.* (1994 ▶). For a discussion of the geometry of tin in diorganotin bis­chelates, see: Ng *et al.* (1987 ▶).
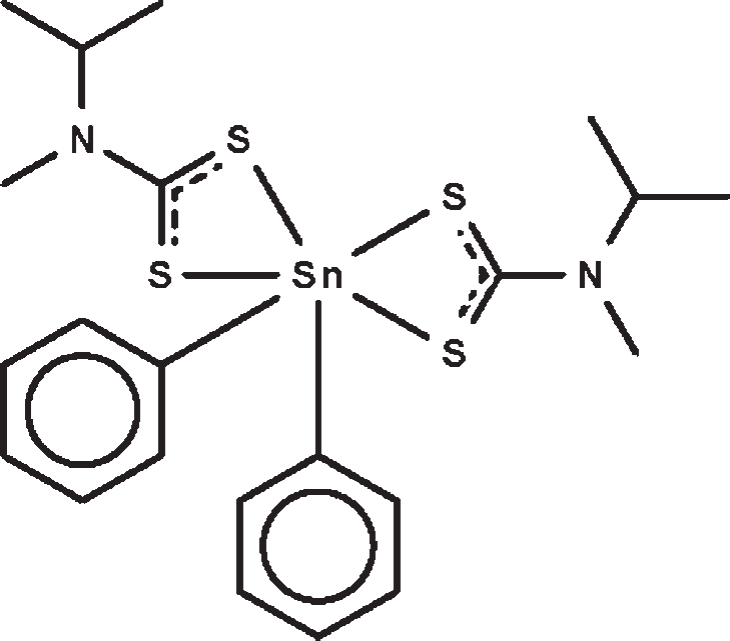


## Experimental

### 

#### Crystal data

[Sn(C_6_H_5_)_2_(C_5_H_10_NS_2_)_2_]
*M* *_r_* = 569.41
Orthorhombic, 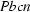

*a* = 18.8797 (10) Å
*b* = 9.2067 (5) Å
*c* = 14.5694 (8) Å
*V* = 2532.4 (2) Å^3^
*Z* = 4
Mo *K*α radiation
μ = 1.35 mm^−1^
*T* = 293 K
0.35 × 0.35 × 0.20 mm

#### Data collection

Bruker SMART APEX diffractometer
Absorption correction: multi-scan (*SADABS*; Sheldrick, 1996 ▶) *T*_min_ = 0.649, *T*_max_ = 0.774
15127 measured reflections
2785 independent reflections
2291 reflections with *I* > 2σ(*I*)
*R*_int_ = 0.022

#### Refinement

*R*[*F* ^2^ > 2σ(*F* ^2^)] = 0.026
*wR*(*F* ^2^) = 0.089
*S* = 1.10
2785 reflections
135 parameters
H-atom parameters constrained
Δρ_max_ = 0.29 e Å^−3^
Δρ_min_ = −0.30 e Å^−3^

### 

Data collection: *APEX2* (Bruker, 2009 ▶); cell refinement: *SAINT* (Bruker, 2009 ▶); data reduction: *SAINT*; program(s) used to solve structure: *SHELXS97* (Sheldrick, 2008 ▶); program(s) used to refine structure: *SHELXL97* (Sheldrick, 2008 ▶); molecular graphics: *X-SEED* (Barbour, 2001 ▶); software used to prepare material for publication: *publCIF* (Westrip, 2010 ▶).

## Supplementary Material

Crystal structure: contains datablocks global, I. DOI: 10.1107/S1600536810007415/ci5039sup1.cif

Structure factors: contains datablocks I. DOI: 10.1107/S1600536810007415/ci5039Isup2.hkl

Additional supplementary materials:  crystallographic information; 3D view; checkCIF report

## Figures and Tables

**Table 1:** Selected bond lengths (Å)

Sn1—C1	2.167 (3)
Sn1—S1	2.5820 (7)
Sn1—S2	2.6910 (8)

**Fig. 1.**
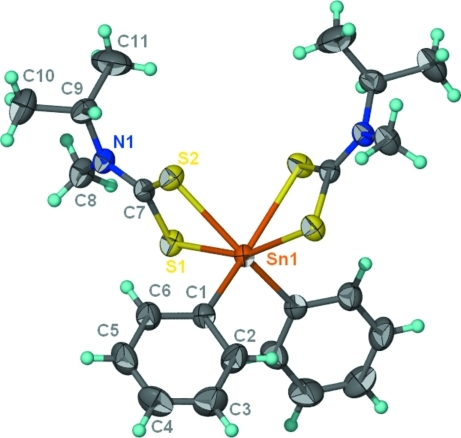
Displacement ellipsoid plot (Barbour, 2001) of [Sn(C6H5)2(C5H10NS2)2] at the 50% probability level. H atoms are drawn as spheres of arbitrary radii. Unlabelled atoms are related to labelled atoms by the symmetry operation (1 - x, y, 1/2 - z).

## supplementary crystallographic information

### Experimental

Diphenyltin dichloride (10 mmol), isopropylmethylamine (10 mmol) and carbon disulfide (10 mmol) were reacted in ethanol (50 ml) at 277 K to produce a white solid. The mixture was stirred for 1 h. The solid was collected and recrystallized from ethanol.

### Refinement

H atoms were placed in calculated positions (C–H = 0.93–0.98 Å) and were included in the refinement in the riding model approximation, with *U_iso_*(H) set to 1.2-1.5*U_eq_*(C).

### Crystal data

**Table:**

[Sn(C_6_H_5_)_2_(C_5_H_10_NS_2_)_2_]	*F*(000) = 1160
*M**_r_* = 569.41	*D*_x_ = 1.493 Mg m^−^^3^
Orthorhombic, *P**b**c**n*	Mo *K*α radiation, λ = 0.71073 Å
Hall symbol: -P 2n 2ab	Cell parameters from 6898 reflections
*a* = 18.8797 (10) Å	θ = 2.4–28.3°
*b* = 9.2067 (5) Å	µ = 1.35 mm^−^^1^
*c* = 14.5694 (8) Å	*T* = 293 K
*V* = 2532.4 (2) Å^3^	Block, colourless
*Z* = 4	0.35 × 0.35 × 0.20 mm

### Data collection

**Table:**

Bruker SMART APEX diffractometer	2785 independent reflections
Radiation source: fine-focus sealed tube	2291 reflections with *I* > 2σ(*I*)
graphite	*R*_int_ = 0.022
ω scans	θ_max_ = 27.5°, θ_min_ = 2.2°
Absorption correction: multi-scan (*SADABS*; Sheldrick, 1996)	*h* = −22→22
*T*_min_ = 0.649, *T*_max_ = 0.774	*k* = −11→11
15127 measured reflections	*l* = −13→18

### Refinement

**Table:**

Refinement on *F*^2^	Primary atom site location: structure-invariant direct methods
Least-squares matrix: full	Secondary atom site location: difference Fourier map
*R*[*F*^2^ > 2σ(*F*^2^)] = 0.026	Hydrogen site location: inferred from neighbouring sites
*wR*(*F*^2^) = 0.089	H-atom parameters constrained
*S* = 1.10	*w* = 1/[σ^2^(*F*_o_^2^) + (0.044*P*)^2^ + 2.0829*P*] where *P* = (*F*_o_^2^ + 2*F*_c_^2^)/3
2785 reflections	(Δ/σ)_max_ = 0.001
135 parameters	Δρ_max_ = 0.29 e Å^−^^3^
0 restraints	Δρ_min_ = −0.30 e Å^−^^3^

### Fractional atomic coordinates and isotropic or equivalent isotropic displacement parameters (Å^2^)

**Table:**

	*x*	*y*	*z*	*U*_iso_*/*U*_eq_	
Sn1	0.5000	0.71348 (3)	0.2500	0.03365 (10)	
S1	0.44810 (4)	0.65957 (8)	0.41039 (4)	0.04395 (18)	
S2	0.40918 (5)	0.48826 (9)	0.24740 (4)	0.04373 (19)	
N1	0.36739 (12)	0.4240 (3)	0.41754 (14)	0.0388 (5)	
C1	0.42071 (15)	0.8630 (3)	0.19862 (19)	0.0404 (6)	
C2	0.44326 (19)	0.9844 (4)	0.1519 (3)	0.0648 (10)	
H2	0.4911	0.9945	0.1382	0.078*	
C3	0.3962 (2)	1.0925 (4)	0.1246 (3)	0.0802 (12)	
H3	0.4128	1.1745	0.0941	0.096*	
C4	0.3258 (2)	1.0776 (4)	0.1429 (3)	0.0722 (11)	
H4	0.2943	1.1505	0.1258	0.087*	
C5	0.3015 (2)	0.9560 (4)	0.1861 (3)	0.0692 (10)	
H5	0.2533	0.9448	0.1969	0.083*	
C6	0.34853 (18)	0.8492 (4)	0.2139 (2)	0.0544 (8)	
H6	0.3314	0.7668	0.2434	0.065*	
C7	0.40355 (14)	0.5130 (3)	0.36386 (17)	0.0356 (5)	
C8	0.36473 (18)	0.4480 (4)	0.51766 (19)	0.0518 (8)	
H8A	0.4088	0.4889	0.5379	0.078*	
H8B	0.3568	0.3571	0.5482	0.078*	
H8C	0.3268	0.5137	0.5319	0.078*	
C9	0.33678 (18)	0.2865 (3)	0.3824 (2)	0.0486 (7)	
H9	0.3324	0.2958	0.3156	0.058*	
C10	0.2637 (2)	0.2584 (5)	0.4203 (3)	0.0784 (12)	
H10A	0.2425	0.1787	0.3880	0.118*	
H10B	0.2351	0.3437	0.4128	0.118*	
H10C	0.2672	0.2350	0.4844	0.118*	
C11	0.3871 (3)	0.1643 (5)	0.4012 (4)	0.1013 (17)	
H11A	0.3723	0.0795	0.3681	0.152*	
H11B	0.3875	0.1436	0.4658	0.152*	
H11C	0.4339	0.1916	0.3818	0.152*	

### Atomic displacement parameters (Å^2^)

**Table:**

	*U*^11^	*U*^22^	*U*^33^	*U*^12^	*U*^13^	*U*^23^
Sn1	0.03059 (17)	0.03725 (15)	0.03310 (15)	0.000	0.00153 (9)	0.000
S1	0.0463 (4)	0.0523 (4)	0.0332 (3)	−0.0086 (3)	0.0028 (3)	−0.0052 (3)
S2	0.0555 (5)	0.0467 (4)	0.0290 (3)	−0.0106 (3)	0.0052 (3)	−0.0015 (3)
N1	0.0427 (13)	0.0454 (12)	0.0282 (10)	−0.0041 (10)	0.0043 (9)	0.0025 (9)
C1	0.0377 (16)	0.0452 (14)	0.0383 (14)	−0.0004 (11)	−0.0031 (11)	−0.0004 (11)
C2	0.050 (2)	0.063 (2)	0.082 (2)	−0.0027 (17)	−0.0015 (18)	0.0269 (19)
C3	0.085 (3)	0.062 (2)	0.093 (3)	0.006 (2)	−0.007 (2)	0.037 (2)
C4	0.072 (3)	0.072 (2)	0.073 (2)	0.029 (2)	−0.021 (2)	0.0082 (19)
C5	0.042 (2)	0.082 (3)	0.084 (3)	0.0168 (18)	−0.0038 (18)	0.008 (2)
C6	0.0450 (19)	0.0591 (19)	0.0591 (19)	0.0038 (15)	0.0025 (15)	0.0092 (16)
C7	0.0346 (14)	0.0408 (13)	0.0315 (12)	0.0029 (11)	0.0014 (10)	0.0024 (10)
C8	0.057 (2)	0.0659 (19)	0.0327 (13)	−0.0092 (16)	0.0060 (13)	0.0025 (13)
C9	0.060 (2)	0.0449 (16)	0.0413 (15)	−0.0084 (13)	0.0040 (14)	0.0043 (12)
C10	0.058 (3)	0.090 (3)	0.087 (3)	−0.026 (2)	0.004 (2)	0.004 (2)
C11	0.103 (4)	0.052 (2)	0.148 (5)	0.016 (2)	−0.014 (3)	−0.014 (3)

### Geometric parameters (Å, °)

**Table:**

Sn1—C1^i^	2.167 (3)	C4—C5	1.364 (6)
Sn1—C1	2.167 (3)	C4—H4	0.93
Sn1—S1^i^	2.5820 (7)	C5—C6	1.386 (5)
Sn1—S1	2.5820 (7)	C5—H5	0.93
Sn1—S2	2.6910 (8)	C6—H6	0.93
Sn1—S2^i^	2.6909 (8)	C8—H8A	0.96
S1—C7	1.728 (3)	C8—H8B	0.96
S2—C7	1.715 (3)	C8—H8C	0.96
N1—C7	1.323 (3)	C9—C10	1.508 (5)
N1—C8	1.476 (3)	C9—C11	1.498 (5)
N1—C9	1.483 (4)	C9—H9	0.98
C1—C2	1.376 (4)	C10—H10A	0.96
C1—C6	1.387 (4)	C10—H10B	0.96
C2—C3	1.392 (5)	C10—H10C	0.96
C2—H2	0.93	C11—H11A	0.96
C3—C4	1.362 (6)	C11—H11B	0.96
C3—H3	0.93	C11—H11C	0.96
			
C1^i^—Sn1—C1	101.13 (15)	C4—C5—H5	119.9
C1^i^—Sn1—S1^i^	99.94 (8)	C6—C5—H5	119.9
C1—Sn1—S1^i^	94.10 (7)	C1—C6—C5	121.1 (3)
C1^i^—Sn1—S1	94.10 (7)	C1—C6—H6	119.4
C1—Sn1—S1	99.94 (8)	C5—C6—H6	119.4
S1^i^—Sn1—S1	157.84 (4)	N1—C7—S2	122.3 (2)
C1^i^—Sn1—S2	159.15 (7)	N1—C7—S1	120.20 (19)
C1—Sn1—S2	92.55 (8)	S2—C7—S1	117.49 (15)
S1^i^—Sn1—S2	94.64 (2)	N1—C8—H8A	109.5
S1—Sn1—S2	67.84 (2)	N1—C8—H8B	109.5
C1^i^—Sn1—S2^i^	92.55 (8)	H8A—C8—H8B	109.5
C1—Sn1—S2^i^	159.15 (7)	N1—C8—H8C	109.5
S1^i^—Sn1—S2^i^	67.84 (2)	H8A—C8—H8C	109.5
S1—Sn1—S2^i^	94.64 (2)	H8B—C8—H8C	109.5
S2—Sn1—S2^i^	79.19 (4)	N1—C9—C10	112.1 (3)
C7—S1—Sn1	88.84 (9)	N1—C9—C11	109.3 (3)
C7—S2—Sn1	85.58 (10)	C10—C9—C11	112.6 (3)
C7—N1—C8	120.6 (2)	N1—C9—H9	107.5
C7—N1—C9	121.7 (2)	C10—C9—H9	107.5
C8—N1—C9	117.1 (2)	C11—C9—H9	107.5
C2—C1—C6	117.3 (3)	C9—C10—H10A	109.5
C2—C1—Sn1	118.2 (2)	C9—C10—H10B	109.5
C6—C1—Sn1	124.4 (2)	H10A—C10—H10B	109.5
C1—C2—C3	121.7 (3)	C9—C10—H10C	109.5
C1—C2—H2	119.2	H10A—C10—H10C	109.5
C3—C2—H2	119.2	H10B—C10—H10C	109.5
C4—C3—C2	119.6 (4)	C9—C11—H11A	109.5
C4—C3—H3	120.2	C9—C11—H11B	109.5
C2—C3—H3	120.2	H11A—C11—H11B	109.5
C3—C4—C5	120.1 (3)	C9—C11—H11C	109.5
C3—C4—H4	120.0	H11A—C11—H11C	109.5
C5—C4—H4	120.0	H11B—C11—H11C	109.5
C4—C5—C6	120.2 (3)		
			
C1^i^—Sn1—S1—C7	−166.32 (12)	C6—C1—C2—C3	2.9 (6)
C1—Sn1—S1—C7	91.61 (12)	Sn1—C1—C2—C3	−173.6 (3)
S1^i^—Sn1—S1—C7	−36.91 (9)	C1—C2—C3—C4	−1.2 (7)
S2—Sn1—S1—C7	2.91 (9)	C2—C3—C4—C5	−1.3 (7)
S2^i^—Sn1—S1—C7	−73.43 (9)	C3—C4—C5—C6	1.9 (6)
C1^i^—Sn1—S2—C7	28.6 (2)	C2—C1—C6—C5	−2.2 (5)
C1—Sn1—S2—C7	−102.64 (12)	Sn1—C1—C6—C5	174.0 (3)
S1^i^—Sn1—S2—C7	163.03 (9)	C4—C5—C6—C1	−0.1 (6)
S1—Sn1—S2—C7	−2.94 (9)	C8—N1—C7—S2	179.1 (2)
S2^i^—Sn1—S2—C7	96.66 (9)	C9—N1—C7—S2	7.8 (4)
C1^i^—Sn1—C1—C2	41.2 (2)	C8—N1—C7—S1	−0.2 (4)
S1^i^—Sn1—C1—C2	−59.7 (3)	C9—N1—C7—S1	−171.5 (2)
S1—Sn1—C1—C2	137.5 (3)	Sn1—S2—C7—N1	−174.7 (2)
S2—Sn1—C1—C2	−154.6 (3)	Sn1—S2—C7—S1	4.59 (14)
S2^i^—Sn1—C1—C2	−88.8 (3)	Sn1—S1—C7—N1	174.6 (2)
C1^i^—Sn1—C1—C6	−135.0 (3)	Sn1—S1—C7—S2	−4.77 (15)
S1^i^—Sn1—C1—C6	124.0 (3)	C7—N1—C9—C10	−139.3 (3)
S1—Sn1—C1—C6	−38.8 (3)	C8—N1—C9—C10	49.2 (4)
S2—Sn1—C1—C6	29.2 (3)	C7—N1—C9—C11	95.2 (4)
S2^i^—Sn1—C1—C6	94.9 (3)	C8—N1—C9—C11	−76.4 (4)

Symmetry codes: (i) −*x*+1, *y*, −*z*+1/2.
